# Sudden Onset and Blinding Spontaneous Direct Carotid-Cavernous Fistula

**Published:** 2011-01

**Authors:** Mohammad-Reza Razeghinejad, Mansooreh Jamshidian Tehrani

**Affiliations:** Ophthalmology Department and Poostchi Eye Research Center, Shiraz University of Medical Sciences, Shiraz, Iran

**Keywords:** Carotid-Cavernous Sinus Fistula, Blindness, Proptosis

## Abstract

**Purpose:**

To report a case of spontaneous direct carotid-cavernous fistula causing abrupt loss of vision.

**Case Report:**

A 50-year-old woman with systemic hypertension but no history of ocular disease developed sudden proptosis, frozen eye, subconjunctival hemorrhage and loss of vision in her left eye over 2 hours. Imaging studies revealed a direct carotid-cavernous fistula. Management for high intraocular pressure was promptly initiated and the patient was referred to a neurosurgery service, but she refused any surgical intervention. Ultimately, she accepted to undergo manual carotid artery compression which resulted in significant reduction in the proptosis, but she lost all vision permanently.

**Conclusion:**

Direct carotid-cavernous fistula can occur spontaneously and should be taken into account in patients with signs suggestive of direct carotid-cavernous sinus fistula even without history of trauma or connective tissue disorder.

## INTRODUCTION

Carotid-cavernous fistula (CCF) is an abnormal communication between the high-pressure carotid arterial system and the low-pressure cavernous venous system manifesting with a classic triad of chemosis, proptosis and bruit.^1^ There are several classifications of CCF based on anatomy, etiology and pathophysiology. Anatomically, CCF is classified as direct and indirect. Direct CCFs are more common, accounting for 60 to 70% of all cases^2^ and may occur following a traumatic tear in the cavernous segment of the internal carotid artery (ICA) or rupture of an aneurysm within this segment. Usual causes of traumatic CCFs include road-traffic accidents, fights and falls which account for approximately 75% of cases.^3^,^4^ Indirect or dural CCFs, which occur spontaneously are abnormal communications between the smaller meningeal arterial branches of the internal or external carotid system and the cavernous sinus.^3^ The exact etiology of indirect CCFs is unknown but pregnancy, systemic hypertension, atherosclerotic vascular diseases, connective tissue disorders (such as Ehlers–Danlos syndrome), minor trauma and iatrogenic mechanisms have been implicated.

ICA wall disruption allows high pressure arterial blood to move directly into the cavernous sinus and ophthalmic veins, leading to venous hypertension. The usual findings are ophthalmic signs and symptoms (proptosis, chemosis, conjunctival injection, and visual loss); cranial nerve pareses; bleeding from the mouth, nose or ears; raised intracranial pressure, and intracranial hemorrhage.^5^

Herein, we report a case of sudden onset spontaneous direct CCF, resulting in blindness in 2 hours.

## CASE REPORT

A 50-year-old woman presented to Ophthalmic Emergency Department with sudden onset proptosis and decreased vision in her left eye of 2 hours duration. She had history of systemic hypertension and was on atenolol 100 mg daily. She had no history of trauma or any ocular disorder.

**Figure 1:**
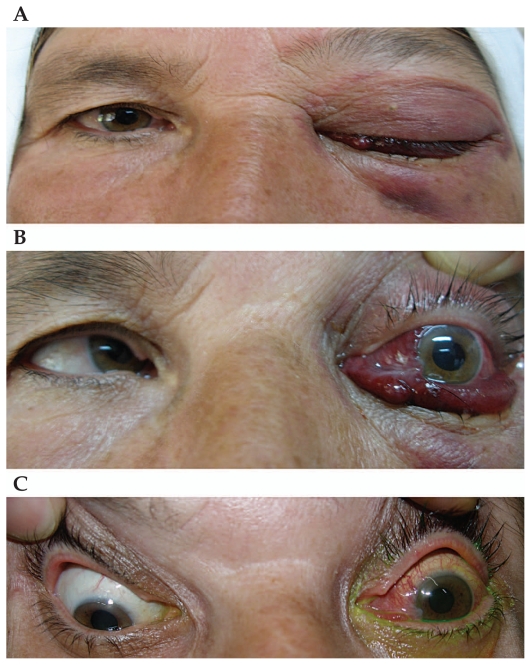
**(A)** Ptosis, periorbital ecchymosis and subconjunctival hemorrhage with protrusion of the conjunctiva in the left eye. **(B)** Left eye proptosis, limitation in ductions, severe subconjunctival hemorrhage and protrusion. **(C)** Two months after initial presentation, proptosis of the left eye was reduced significantly but conjunctival vessels were still hyperemic.

On ophthalmologic examination, the right eye was normal but the left eye had no light perception, intraocular pressure (IOP) of 50 mmHg, profound relative afferent pupil defect, severe proptosis, frozen eye, subconjunctival hemorrhage and periorbital ecchymosis (Fig. 1A and 1B). A prominent bruit was heard over the globe. Intravenous mannitol was initiated and lateral canthotomy was performed to control the raised IOP.

**Figure 2:**
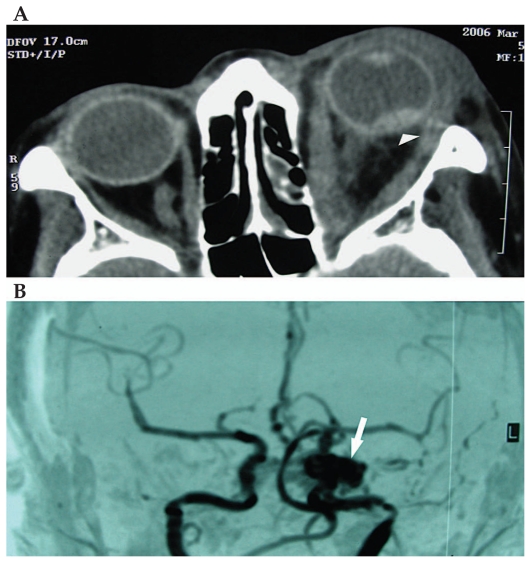
**(A)** Axial computed tomography scan of the orbit: the left eye is severely proptotic, extraocular muscles are enlarged and an intraocular hemorrhage is noted (arrowhead). **(B)** Magnetic resonance angiographic view of a spontaneous high flow carotid-cavernous fistula on the left side (arrow).

Orbital computed topography (CT) scan revealed proptosis, a dilated superior ophthalmic vein, enlarged orbital muscles, and intraocular hemorrhage (Fig. 2A). Magnetic resonance angiography (MRA) and selective carotid angiography showed a high flow arteriovenous shunt (Fig. 2B). The patient was then transferred to the neurosurgery department for further management, but she refused to undergo surgery because of possible complications. Therefore, she was recommended to undergo manual carotid compression. Two months later, she had mild proptosis and congested conjunctival vessels, but visual acuity was unchanged at no light perception (Fig. 1C).

## DISCUSSION

Spontaneous CCFs are extremely rare, most of them are indirect and supplied by one or more meningeal branches of the internal or external carotid artery or both. Although occasionally associated with systemic connective tissue disorders such as Ehlers-Danlos syndrome, these aneurysms are more often caused by rupture of intracaverneous ICA aneurysms. Yu et al^6^ have reported nine patients with direct spontaneous CCF, which is the highest number of subjects reported in the literature. Internal carotid angiograms recorded during the early arterial phase revealed aneurysms located in the intracavernous portion of the ICA in eight patients, suggesting them as culprits for CCFs. In our patient no evidence for an aneurysm was detected on neuroimaging. Das et al^2^ reported 3 cases of spontaneous indirect CCFs and stated that advanced age, menopause, hypertension and childbirth may affect some pre-existing subclinical lesions in the arteriolar system such as aneurysms, leading to spontaneous CCFs.

The clinical presentation of CCFs depends on the degree of shunting and the route of venous drainage.^1^,^2^ The clinical picture of direct CCFs include sudden onset and pulsatile exophthalmos, conjunctival congestion, chemosis, subconjunctival hemorrhage, ophthalmoplegia, and increased IOP over several days. Diplopia may result from ocular motor nerve pareses, orbital congestion, or both mechanisms. There may be significant periorbital or retro-ocular discomfort or pain, initially suggesting an inflammatory process or even the Tolosa–Hunt syndrome. Increased episcleral venous pressure may lead to high IOP which can occasionally get dramatic. Angle-closure glaucoma may develop from elevated orbital venous pressure, congestion of the iris and choroid, and forward displacement of the iris-lens diaphragm. Ophthalmoscopic abnormalities include optic disc swelling, venous stasis retinopathy with intraretinal hemorrhages, central retinal vein occlusion, proliferative retinopathy, retinal detachment, vitreous hemorrhage, and choroidal folds, effusion and detachment.^3^

Indirect carotid-cavernous fistulae are slow-flow shunts as compared to direct fistulae; they have a gradual onset and milder symptoms. In least severe cases, there is redness of one, or rarely, both eyes caused by dilation and arterialization of both conjunctival and episcleral veins. The appearance of the eye in these cases may suggest conjunctivitis, episcleritis, thyroid eye disease, orbital pseudotumor, orbital cellulitis, spheno-orbital meningioma, or the Tolosa–Hunt syndrome. However, a careful examination of the dilated vessels usually demonstrates a typical tortuous corkscrew appearance that is virtually pathognomonic of a dural CCF.^3^

Physical findings in nine reported cases with direct spontaneous CCF by Yu et al^6^ were cranial bruits in all patients, exophthalmos in eight, decreased vision in three, 3^rd^ and 4^th^ cranial nerve palsy with complete ptosis in one, and 6^th^ cranial nerve palsy and paresis of the extremities in one subject. The only patient who had no proptosis presented with recurrent epistaxis.

Some patients may not display the typical signs of CCF; in these subjects early suspicion by the eye care provider is of paramount importance. A high index of suspicion is the key factor in the diagnosis of CCF without a history of trauma or collagen vascular disorder. CT and MRI studies help to detect proptosis, increased extraocular muscle size and a dilated or thrombosed superior ophthalmic vein. Selective angiography is essential for confirming the diagnosis.

Unlike direct CCFs, most indirect CCFs improve spontaneously and often all clinical manifestations resolve without vascular intervention. However, patients with intractable headache, visual deterioration, refractory IOP, diplopia, or intolerable cosmetic deformity are considered for endovascular treatment.^7^ If left untreated, as many as 90% of patients with direct CCFs may lose vision while 20–30% of patients with indirect types will do so. Patients generally have good prognosis after treatment.^8^ The traditional approach to CCFs include transarterial embolization using liquid agents, particularly n-butyl cyanoacrylate (n-BCA). However, the multiplicity of arterial feeders and the low success rate in occluding indirect CCFs by the arterial route has led to a preference for transvenous embolization. Direct CCFs are always treated with endovascular methods using detachable coils or liquid embolic agents delivered transarterially or transvenously. In rare cases occlusion of the ICA is required. The success rate in expert hands is 85%–99% for closing direct fistulae and 70%–78% for closing indirect types.^5^

Our patient did not accept the risks of surgical intervention, therefore she underwent manual carotid compression. In this modality, while the patient sits in a chair or lies down on a table, the carotid artery and jugular vein are compressed with the contralateral hand for a period of 10 seconds, 4–6 times per hour.^8^ Carotid compression has been reported to be successful in closure of 17% of direct CCFs and 30% of indirect CCFs.^5^ However, manual carotid compression is contraindicated in patients with hypersensitive carotid sinus syndrome, atherosclerotic stenosis, ulceration of the cervical carotid artery, and history of cerebral ischemia. Moreover, compression over the carotid artery may cause bradycardia and shock because of carotid sinus compression.^5^

This report indicates that the clinician should not overlook the possibility of direct spontaneous CCF in patients without any history of trauma or connective tissue disorder who present with an acute clinical picture consistent with CCF.
